# Effects of Buffalo Milk and Cow Milk on Lipid Metabolism in Obese Mice Induced by High Fat

**DOI:** 10.3389/fnut.2022.841800

**Published:** 2022-04-26

**Authors:** Maocheng Jiang, Zitong Meng, Zhiqiang Cheng, Kang Zhan, Xiaoyu Ma, Tianyu Yang, Yinghao Huang, Qi Yan, Xiaoxiao Gong, Guoqi Zhao

**Affiliations:** ^1^College of Animal Science and Technology, Institute of Animal Culture Collection and Application, Yangzhou University, Yangzhou, China; ^2^Institutes of Agricultural Science and Technology Development, Yangzhou University, Yangzhou, China; ^3^Joint International Research Laboratory of Agriculture and Agri-Product Safety, Yangzhou University, Ministry of Education of China, Yangzhou, China

**Keywords:** buffalo, milk, metabonomic, lipid metabolism, inflammatory

## Abstract

The aim of this study was to evaluate the effects of buffalo milk and cow milk on lipid metabolism in obese mice. Milk composition analysis showed fat, protein, and total solid content in buffalo milk was higher than cow milk, while the lactose content of buffalo milk was lower than cow milk. After milk metabolite extraction and LC-MS/MS analysis, differential metabolites were mainly enriched in “linoleic acid metabolism pathways,” “pentose and glucuronate interconversion pathways,” and “metabolism of xenobiotics by cytochrome P450 pathways.” We fed three groups of C57BL/6J mice (*n* = 6 per group) for 5 weeks: (1) high-fat diet group (HFD group); (2) high-fat diet + buffalo milk group (HBM group); and (3) high-fat diet + cow milk group (HCM group). Our results showed that body weight of mice was significantly decreased in HBM and HCM groups from 1 to 4 weeks compared with the HFD group. The mRNA expression of ACAA2, ACACB, and SLC27A5 genes involved in the lipid metabolism in liver tissue were significantly elevated in HCM group, relatively to HFD and HBM group. In addition, the adipocyte number, size and lipid accumulation in the liver were significantly decreased in HCM group compared with the HFD group by H&E staining and oil red O staining, but was not change in HBM group. The mRNA levels of TNF-α and IL-1β inflammatory genes were significantly increased in HBM group, relatively to HFD and HCM group, which is consistent with results from inflammatory cell infiltration and tissue disruption by colon tissue sections. In conclusion, dietary supplementation of cow milk has beneficial effects on loss of weight and lipid metabolism in obese mice.

## Introduction

The complexity of obesity as a disease increases the susceptibility of obese individuals to develop other metabolic diseases, such as diabetes, metabolic syndrome, and cardiovascular diseases. A high fat diet will cause lipid metabolism disorder and fat accumulation in the mouse body, leading to causing obesity (1). The liver is an important organ of lipid metabolism. The lipid metabolism disorder and severe fat infiltration occurred simultaneously in mice fed a high fat diet, resulting in fatty liver (2). Moreover, high-fat diet intake can also change the expression of genes related to metabolic signals, affecting the normal metabolic function of the body (3). The accumulation of white adipose tissue in the body can cause the gradual accumulation and infiltration of macrophages, leading to chronic inflammation (4). Previous studies have found that lipid metabolic diseases may be related to pro-inflammatory responses (5). Long term consumption of high-fat foods also increased the proportion of intestinal pathogens (6). Flora disorder can cause changes in intestinal mucosal tight junction protein, and increase intestinal permeability to induce the absorption of fatty polysaccharides and pro-inflammation response (7). It is now widely believed that the intake of a high-fat diet is the most important factor in inducing metabolic syndrome.

Dairy products are an important part of people’s diets. A wide variety of dairy products contain high-quality protein, various trace elements, and functional components, including milk calcium, whey, polar lipids, and conjugated linoleic acid (8, 9). Indeed, milk protein composed of 80% casein and 20% whey protein are widely perceived as a major protein source (10). In addition, whey protein contains a variety of biologically active components, which can significantly relieve signs of disease (11). Dietary calcium also plays a key role in regulating energy metabolism and is easily absorbed by the body (12). Increasing dietary calcium intake reduces the transcription of fatty acid synthase in adipocytes, causing decreased lipogenesis and insulin secretion (13). Conjugated linoleic acid, known as an important source of unsaturated fatty acid in diet, mainly exists in the dairy products of ruminants. Previous study has demonstrated that dairy products mainly contain levels of *cis*-9 and *trans*-11 isoform of conjugated linoleic acid (14). The obese patients who were fed a diet supplemented with conjugated linoleic acid have a significant reduction in body fat content for 12 weeks (15). In addition to conjugated linoleic acid, isooleic acid is also found in dairy and meat products of ruminants. Studies have reported that isooleic acid has regulation effect on blood-lipid (16). A growing body of research now shows the beneficial effects of milk on improving lipid profiles in blood and liver (17, 18). In recent years, attention has been paid to the potential beneficial effects of milk proteins on risk markers of metabolic syndrome. Previous studies have only reported on the milk fat content of buffalo milk and dairy milk. However, the effects of buffalo milk and cow milk on lipid metabolism in mice induced by high fat remains not reported.

We hypothesized that buffalo milk and cow milk improves lipid metabolism in obese mice induced by high fat. Therefore, objective of this study was to investigate the effects of buffalo milk and cow milk on lipid metabolism in mice induced by high fat.

## Materials and Methods

### Animal Experiment

The animal experiment was approved by the Institutional Animal Care and Use Committee of Yangzhou University, and the study was conducted at Yangzhou University (Yangzhou, China). Eighteen 8-week-old male C57BL/6J mice were obtained from the Comparative Medicine Animal Center of Yangzhou University. Following a week-long acclimation period, principle of near body weight was randomly divided into three groups (*n* = 6 animals per high-fat diet + 0.8 mL normal saline as HFD group (60% fat diets), *n* = 6 animals per high-fat diet + 0.4 mL buffalo milk + 0.4 mL normal saline as HBM group, *n* = 6 animals per high-fat diet + 0.8 mL cow milk as HCM group). For equal protein and energy in HBM group and HCM group was diluted with normal saline. The HFD in this study contained 60% fat (TrophicDiet, Nantong, China). All the mice were housed under standard laboratory conditions (20–22°C; light/dark cycle for 12 h: 12 h) and at least on their diets for 5 weeks. The body weight of each mouse was recorded weekly. After 5 weeks, the serum and tissue samples were collected and stored for further studies.

### Analysis of Milk Composition

Milk samples were collected from three consecutive milking (6:00, 13:00, and 20:00). Each sample was placed in tubes with a preservative (0.05%, Benzoic acid) and submitted to Dairy One Cooperative Inc. (Shanghai, China) for analysis of milk related indicators.

### Milk Metabolite Extraction and LC-MS/MS Analysis

The metabolite in buffalo and cow milk were extracted as described previously (19, 20). In brief, 100 μL of each sample is mixed with 400 μL of precipitant (Methanol: acetonitrile = 2: 1, V/V), vibrated and sonicated, then settled at -20°C for 2 h and finally centrifuged (4,000 rpm, 30 min, 4°C). Each sample was placed in tubes submitted to BGI Cooperative Inc. (BGI, Shenzhen, China) for analysis of LC-MS/MS analysis (Waters, 2777C UPLC system and Xevo G2-XS QTOF, United Kingdom).

### Serum Analysis

At week 5 of intervention, mice were briefly food deprived (between 3 and 8 h starting at 06:00) prior to tissue and blood collection as described in detail (19). Serum glucose, triglyceride (TG), apolipoprotein A1 (ApoA1), and apolipoprotein B (ApoB) levels were measured using the commercially available assay kits (Rongsheng, Shanghai, China).

### Gene Expression Analysis by Real-Time PCR

Total RNA was extracted and purified from colon and liver tissues using the Trizol tissue kit according to the manufacturer instructions (Tiangen, Beijing, China). Reverse transcription (RT) was performed using an RT Kit (Takara, Beijing, China). RT reaction mixtures contained 1 μg total RNA and 1× PrimeScript RT Master Mix in a final volume of 20 μL, and were conducted for 15 min at 37°C. Reverse transcriptase was inactivated by heating to 85°C for 5 s. Quantitative RT-PCR (qRT-PCR) assays were performed using SYBR Premix Ex Taq TM II Kit (Vazyme, Nanjing, China). The qRT-PCR reaction mixture contained 1 × SYBR Premix Ex Taq TM II, 0.4 μM each forward and reverse primers, and 2 μL cDNA templates in a final volume of 20 μL. Reactions were performed as follows: initial denaturation at 95°C for 30 s, followed by 40 cycles at 95°C for 5 s and 60°C for 30 s. The primers used are listed in Table 1 and were synthesized by Suzhou Genewiz Biological Co. (Genewiz, Suzhou, China). These mouse primers were selected from the Primer Bank developed by the Massachusetts General Hospital and Harvard Medical School (21). The relative expression levels of target genes were normalized to those of GAPDH and calculated using the 2^–Δ^ ^Δ^ *^CT^*.

**TABLE 1:** Real-time PCR primers.

Gene	Gene name	Primer sequence (5′-3′)
ACTA2	Actin alpha 2	F: CGAAACCACCTATAACAGCATCA R: GCGTTCTGGAGGGGCAAT
CPT2	Carnitine palmitoyltransferase 2	F: CCTGCTCGCTCAGGATAAACA R: GTGTCTTCAGAAACCGCACTG
ACOX3	Acyl-CoA oxidase 3, pristanoyl	F: CAGAATGGTGTGCTAGAGCGT R: AGCCTGTCGGCTACAGATTTG
ACAA2	Acetyl-CoA acyltransferase 2	F: CTGCTACGAGGTGTGTTCATC R: TCCAAAGGGTGTTCGCTTCG
ACACB	Acetyl-CoA carboxylase β	F: TTCTGAATGTGGCTATCAAGACTGA R: TGCTGGGTGAACTCTCTGAACA
ACAT2	Acetyl-CoA acetyltransferase 2	F: CCCGTGGTCATCGTCTCAG R: GGACAGGGCACCATTGAAGG
SCD1	Stearoyl-CoA desaturase 1	F: GATAGAGCAAGTCCCCGCTG R: CCTGCATTAACCCCCTTCAC
SLC27A5	Solute carrier family 27, member 5	F: GGAGGTGGTGATAGCCGGTAT R: TGGGTAATCCATAGAGCCCAG
IL-1β	Interleukin-1β	F: TCCATGAGCTTTGTACAAGGA R: AGCCCATACTTTAGGAAGACA
TNF-α	Tumor necrosis factor-alpha	F: TAGCCAGGAGGGAGAACAGA R: TTTTCTGGAGGGAGATGTGG
β-actin	Actin, β	F: CCTTCTTGGGTATGGAATCCTGTG R: CAGCACTGTGTTGGCATAGAGG

*F, Forward Primer; R, Reverse Primer.*

### Histological Analysis of the Liver and Inflammation of the Colon

#### Hematoxylin and Eosin Staining

Small pieces of colon tissue were fixed in 10% phosphate-buffered saline (PBS) formalin and embedded in paraffin wax for hematoxylin and eosin (H & E) staining. After dehydration, the slices were sealed with neutral gum. The histology slides were performed using an optical microscope (Olympus, Olympus Optical Ltd., Tokyo, Japan), with images being captured at a final magnification of 200×. The adipocyte area was measured using image analysis software (Image J 1.47, National Institutes of Health, United States).

#### Oil Red O Staining

Oil red O staining was performed after small pieces of liver tissue was immersed in 60% isopropanol. Finally, these slides were sealed with glycerin gelatin. The histology slides were performed using an optical microscope (Olympus, Olympus Optical Ltd., Tokyo, Japan), with images being captured at a final magnification of 200×. The fatty droplets area in hepatocytes was observed by oil red O staining and image analysis software (Image J 1.47, National Institutes of Health, United States).

### Statistical Analysis

MetaboAnalyst 5.0 (22) was used for integrative metabolomics data and pathway analysis. Data are presented as the means ± standard error (SEM). Significant differences were determined by one-way ANOVA and Tukey’s multiple comparison tests. The analysis was conducted using the SPSS Statistics software, version 20.0 (IBM Corp., Armonk, NY, United States). Different letters represent significant differences (*P* < 0.05), while the same letters represent no significant differences (*P* > 0.05).

## Results

### Analysis of Milk Metabolites in the Buffalo and Cow

#### Analysis of Milk Composition

The indexes of fat, protein, and total solid content in buffalo milk were higher than cow milk (*P* < 0.01). However, the lactose content of buffalo milk was lower than that of cow milk (*P* < 0.05; Table 2).

**TABLE 2:** Comparisons of milk composition between buffalos and cows (*n* = 4).

Composition	Buffalo milk	Cow milk	SEM	*P*-value
Lactose (%)	2.22	5.20	0.44	<0.01
Fat (%)	8.68	4.07	1.03	0.017
Protein (%)	9.44	3.25	0.71	<0.01
Total solid content (%)	18.88	12.75	1.29	<0.01

#### Analysis of Enrichment Metabolic Pathways in Buffalo and Cow Milk

**FIGURE 1:**
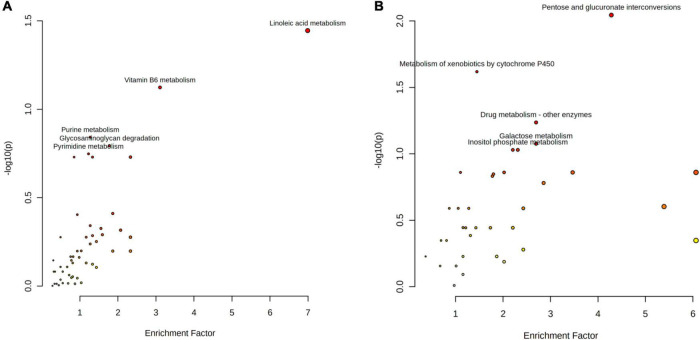
The metabolome view map of significant metabolic pathways identified in buffalo and cow milk. **(A)** Positive ionization modes; **(B)** negative ionization modes.

In order to investigate the function of differential metabolites, differential metabolites pathways were analyzed by metaboanalyst 5.0 (Figure 1). Multiple metabolic pathways were obtained when significantly different metabolites were imported into KEGG. Of these, a comprehensive analysis of the *P*-value and Gamma P showed that differential metabolites were mainly enriched in “linoleic acid metabolism pathways,” “pentose and glucuronate interconversion pathways,” and “metabolism of xenobiotics by cytochrome P450 pathways” (*P* < 0.05; Tables 3, 4).

**TABLE 3:** Analysis of enriched metabolic pathways in buffalo and cow milk for positive ion mode.

Pathway	Hits*^a^*	*P*-value	Gamma *P^b^*
Linoleic acid metabolism	4	0.03	0.04
Vitamin B6 metabolism	5	0.07	0.05
Purine metabolism	28	0.14	0.049
Pyrimidine metabolism	14	0.18	0.05
Alpha-Linolenic acid metabolism	7	0.19	0.06
Arginine and proline metabolism	15	0.68	0.13
Biosynthesis of unsaturated fatty acids	15	0.68	0.13
Amino sugar and nucleotide sugar metabolism	27	0.75	0.13
Glycosaminoglycan degradation	10	0.16	0.06
Galactose metabolism	21	0.97	0.28
Fatty acid biosynthesis	4	0.63	1

*^a^Hits represents the number of metabolites in one pathway. ^b^Gamma P indicates the statistical P-values that were further adjusted using the Gamma method for multiple tests. The same as below.*

**TABLE 4:** Analysis of enriched metabolic pathways in buffalo and cow milk for negative ion mode.

Pathway	Hits	*P*-value	Gamma *P*
Fatty acid biosynthesis	5	0.53	1
Fructose and mannose metabolism	3	0.36	1
Pentose and glucuronate interconversions	4	<0.01	0.02
Vitamin B6 metabolism	5	0.07	0.05
Metabolism of xenobiotics by cytochrome P450	9	0.02	0.02
Arginine and proline metabolism	10	0.41	0.07
Biosynthesis of unsaturated fatty acids	16	0.17	0.03
Galactose metabolism	8	0.08	0.03
Drug metabolism–other enzymes	7	0.06	0.03
Linoleic acid metabolism	4	0.45	1
Phenylalanine, tyrosine, and tryptophan biosynthesis	1	0.14	1
Amino sugar and nucleotide sugar metabolism	4	0.45	1
Glycerolipid metabolism	1	0.14	1

### Effects of Buffalo and Cow Milk on Body Weight in Mice Fed by High Fat Diet

**FIGURE 2:**
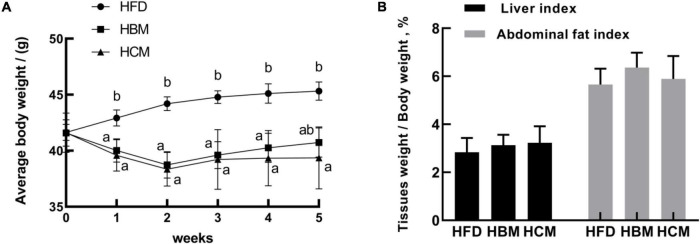
Effects of buffalo and cow milk on body weight in high-fat diet fed mice. **(A)** Average body weights from mice of each group; **(B)** adipose tissues and liver index (tissues weight/body weight). *^a^*–*^b^* Means with different superscripts are significantly different (*n* = 6).

To investigate the effect of different milk types on body weight and fat deposition, we established an obese mouse model by feeding mice a high-fat diet. The body weight was measured every week and there was a significant difference between the HFD, HBM, and HCM group. Compared with the HFD group, the HBM and HCM group had a significantly decreased body weight from 1 to 4 weeks (*P* < 0.05; Figure 2A). In the fifth week, the weight was also significantly lower in the HCM group in relatively to HFD group (*P* < 0.05). In addition, abdominal fat and liver tissue were analyzed. The index of abdominal fat and liver tissue from mice treated with buffalo and cow milk had no profound alter compared to the HFD group (*P* > 0.05; Figure 2B). These results indicated that buffalo and cow milk have a beneficial effect on the health in mice fed by high-fat diet.

### Effects of Buffalo and Cow Milk on Serum Lipid Profile in Mice Fed by High Fat Diet

Studies have found that obesity is commonly accompanied by high serum glucose and high triglyceride associated with several metabolic diseases in obese mice. To investigate whether buffalo and cow milk improve serum metabolic parameters in obese mice, concentrations of serum glucose, TG, ApoA1, and ApoB were analyzed (Table 5). Serum glucose and TG concentrations were slightly decreased in the HBM-fed mice compared to the HFD-fed and HCM-fed mice, but there was no significant difference. Moreover, there were no marked changes in serum apoA1 and ApoB concentrations between the HBM-fed and HCM-fed mice.

**TABLE 5:** Effects of buffalo and cow milk on serum lipid profile in high-fat diet fed mice (*n* = 6).

Index	Treatment group			*SEM*	*P*-value
	HFD	HBM	HCM		
Triglyceride, mmol/L	1043.79	988.91	1023.94	19.98	0.554
Glucose, mmol/L	1542.84	1321.34	1431.14	65.90	0.415
ApoA1, mmol/L	0.54	0.51	0.51	0.08	0.446
ApoB, mmol/L	0.44	0.41	0.42	0.006	0.373

### Effects of Buffalo and Cow Milk on the Expression Level of Genes Related to Hepatic Lipid Metabolism in Mice Fed by High Fat Diet

To further elucidate the buffalo and cow milk on the regulation of genes related to hepatic lipid metabolism, we examined the mRNA expression of genes related to lipid metabolism using qRT-PCR (Table 6). These results showed that the ACAA2, ACACB, and SLC27A5 genes were significantly increased after HCM group intervention in mice fed by high fat diet compared to HFD group. Meanwhile, the ACAA2 gene expression in HBM group was significantly higher than HFD group (*P* < 0.05; Table 6). These results suggested that cow milk can eliminate the fatty liver in mice fed by high fat diet and regulate the balance in hepatic lipid metabolism.

**TABLE 6:** Effects of buffalo and cow milk on the expression level of genes involved in the hepatic lipid metabolism in high-fat diet fed mice (*n* = 6).

Gene	Treatment group			SEM	*P*-value
	HFD	HBM	HCM		
Alpha 2-actin	1.20	1.76	0.96	0.21	0.288
CPT2	1.25	2.02	1.91	0.20	0.192
ACOX3	1.10	1.12	1.76	0.15	0.116
ACAA2	1.04^a^	1.72^b^	2.55^c^	0.18	<0.01
ACACB	1.16^a^	1.14^a^	2.38^b^	0.21	<0.05
ACAT2	1.03	1.24	1.68	0.14	0.168
SCD1	1.05	0.76	1.03	0.09	0.342
SLC27A5	1.06^a^	1.16^a^	1.97^b^	0.16	<0.05

*Within the same line, different letters represent significant differences (P < 0.05), while the same letters represent no significant differences (P > 0.05).*

### Effects of Buffalo and Cow Milk on the Morphology of Liver Tissues in Mice Fed by High Fat Diet

**FIGURE 3:**
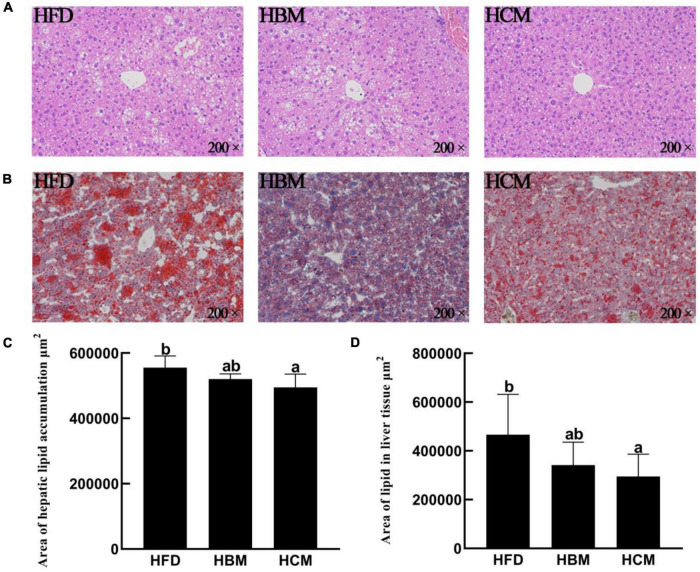
Effects of buffalo and cow milk on hepatic lipid accumulation in mice fed by high fat diet. **(A)** H & E staining of the liver tissues from representative mice of each group (200×); **(B)** oil red O staining of the liver tissues from representative mice of each group (200×); **(C)** calculated value for lipid area of **(A)**; **(D)** calculated value for lipid area of **(B)**.

To explore the effect of buffalo and cow milk on hepatic lipid accumulation in mice induced by high fat diet, we performed H&E and Oil red O staining on the liver tissues (Figures 3A,B). As shown in Figures 3C,D, we found that area of lipid accumulation significantly decreased by cow milk intervention compared with HFD group (*P* < 0.05), but was not change by buffalo milk intervention.

### Effects of Buffalo and Cow Milk on Morphology of Colon Tissues and Expression Level of Genes Related to Pro-inflammation Responses in Mice Fed by High Fat Diet

**FIGURE 4:**
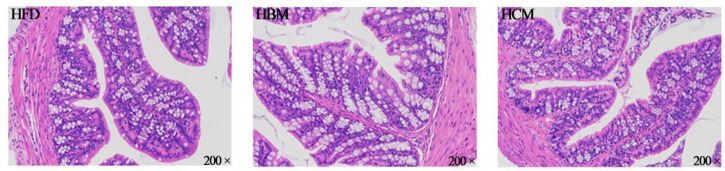
Effects of buffalo and cow milk on morphology of colon tissues in mice fed by high fat diet. H & E staining of the colon morphology from representative mice of each group (200×).

**FIGURE 5:**
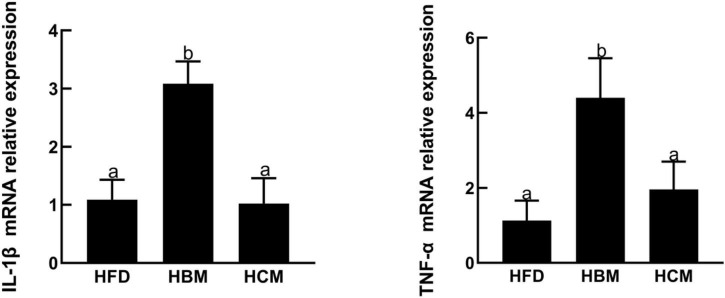
Effects of buffalo and cow milk on expression level of genes related to pro-inflammation responses in mice fed by high fat diet.

To explore the effect of buffalo and cow milk on morphology of colon tissues in mice with high fat diet treatment, the colon H&E staining was performed. The HCM group had no significant change in the morphology of colon compared to HFD group, but morphology of colon was damaged by buffalo milk (Figure 4). Consistently, the IL-1β and TNF-α expression levels of genes involved in pro-inflammatory responses were significantly upregulated by buffalo milk intervention in relatively to HFD group (*P* < 0.05; Figure 5).

## Discussion

Dietary active substance may exert a key role in relieving the comorbidities of obesity. The protein and fat of milk are the most important nutrients, and are important indicators to measure milk quality (23). The content, source, and composition of feed nutrients in dairy cow diet have a significant effect on dairy quality. Our results showed that there were significant differences between buffalo and cow in the indexes of lactose, fat, protein, and total solid content by analysis of milk composition. This difference may be caused by differences in their dietary structure, species, and living areas. These differential metabolites are widely distributed in pathways involved in the milk fat synthesis by analysis of metabolomic, including linoleic acid metabolism, indicating that milk fat synthesis exhibited differential in buffalo and cow milk. We hypothesized that buffalo and cow milk can regulate the lipid metabolism balance and relieve the obese in mice fed by high fat diet due to containing the unsaturated fatty acid in buffalo and cow milk.

In our study, mice body weight was significantly decreased in HBM and HCM compared to HFD group. Studies have demonstrated a relationship between milk intake and body weight in children and adolescents (9, 24, 25). Milk products are an important source of protein. High-protein diets have been shown to induce more weight loss than conventional diets in overweight and obese adults. In addition, dietary protein has been found to be effective in treating overweight and obesity (26–28). Many studies have shown an inverse association between milk intake and the risk of metabolic syndrome (29, 30). Milk composition’s contribution to these effects is unclear, but it has been suggested that the protein content in dairy products is more important than calcium for weight loss in overweight adults (31). In addition, milk contains a large amount of amino acids, and both leucine and isoleucine can reduce weight gain in animals and *in vivo* studies (32, 33). Previous study had reported that whey protein can relieve weight in overweight adults (34). Our data demonstrated that the high protein content of milk plays a key role in weight loss.

At present, there are many animal studies on obesity. Lee’s index, as an index reflecting the degree of obesity, was first considered to be suitable for the evaluation of mouse obesity model (35). On the one hand, Lee’s index has a significant positive correlation with content of fat in body, which can be used to evaluate the degree of obesity in experimental animals. On the other hand, the degree of individual obesity should include not only the increase of body weight, but also the increase of body fat (36). Therefore, this study verified the results of three groups from body weight, liver weight, and fat weight. Many scholars believe that calcium in milk is responsible for decreased blood lipid. Some scholars have studied the relationship between calcium content of dairy products and milk fat on serum lipid indicators (37). These results showed that milk containing high calcium reduced total cholesterol and LDL-C levels and increased HDL/LDL value in relatively to milk with low calcium, but had no significant effect on HDL-C, indicating that a high milk calcium diet reduced the risk of atherosclerosis, and a high calcium milk diet increased fat excretion in feces. Obesity can lead to dyslipidemia in patients, which is mainly manifested as abnormal indicators of TG, HDL, cholesterol, and LDL (38, 39). Many studies have found an association of ApoA1 and ApoB in obesity and metabolic syndrome (40). In present study, concentrations of serum glucose, TG, ApoA1, and ApoB were not altered in three groups. Although buffalo and cow milk had no profound effect in the serum indicators related to lipid metabolism, it may exert a vital role in the others metabolic organs, such as liver.

Previous studies have shown that dairy products reduce the risk of metabolic syndrome by reducing obesity and blood lipids, and also indirectly suggest that some components from dairy products improve liver fat disease and damage (41). Lipid metabolism is a complex process involved in the regulation of multi-factor, multi-step, and multi-protein/gene (42). The liver is the main organ of lipid metabolism and plays an important role in lipid oxidation, absorption, synthesis, and other metabolic processes. Fat accumulation and oxidation in liver are closely related to the expression level of genes related to fat formation and metabolism. It has been reported that CPT2, ACOX3, ACAA2, SCD1, ACACB, ACAT2, and SLC27A5 are key genes in fatty acid metabolism (43–45). The ACAA2, ACACB, and SLC27A5 genes were significantly elevated after HCM group intervention compared to HFD group. Meanwhile, the ACAA2 gene expression in HBM group was significantly higher than HFD group. These results suggested that cow milk can improve the fatty liver in mice fed by high fat diet and regulate the balance in hepatic lipid metabolism.

The liver is an important organ which maintains the balance of lipid metabolism in the body, and the disorder of liver lipid metabolism will cause excessive fat accumulation in liver tissues, resulting in systemic inflammation and fatty liver. Studies have found that prolonged consumption of HFD causes lipid accumulation in liver and adipose tissue, and obesity is often characterized by an increase in number and size of fat cells (46). High fat diets have been recognized as one of the important factors in the pathogenesis of non-alcoholic fatty liver disease and metabolic syndrome. The liver tissues of HFD mice showed diffuse deposition of fat droplets using H & E and oil red O staining, but there was no obvious local necrosis of liver cells, indicating that high fat feeding can trigger the formation of typical fatty liver. Moreover, buffalo and cow milk can inhibit the excessive accumulation of fatty liver caused by high fat diet, and significantly reduce the number and size of fat droplets in the liver tissues, suggesting that buffalo and cow milk can relieve the fatty liver caused by high fat diet and control the balance of lipid metabolism in liver.

Obesity is characterized by chronic low-level activation of the innate immune system and macrophage-induced metabolic inflammation. The interaction between adipocytes and macrophages plays an important role in the regulation of immune responses. Studies have suggested that obesity is associated with chronic inflammation of many tissues, including the liver, adipose tissue, and intestines (47, 48). The gastrointestinal immune system is the largest secondary immune organ in the human body, and its development is influenced by milk derived peptides during the neonatal period, which enhance immune cell function by stimulating the secretion of antibodies, inflammatory cytokines, or specific enzymes, thereby promoting immune regulation (49, 50). Milk is one of the main sources of dietary protein, in which casein can alleviate the symptoms of intestinal inflammation. Adipose tissue is both the source and target for pro-inflammatory cytokines, such as TNF-α and IL-6 (51). Milk contains milk polar lipids that may play an important role in maintaining the integrity of the gastrointestinal barrier and influencing systemic inflammation and lipid metabolism (5). Previous studies have shown that obesity can lead to the pro-inflammation response and change the morphology of intestinal (52, 53). The morphology of intestine directly reflects the health state of the intestine (54), which is mainly determined by the integrity of villi and degree of inflammatory cell infiltration. In this study, morphology of colon tissues was not damaged compared to HFD group. However, HBM group had damage of varying degrees in morphology of colon tissues and the expression levels of genes related to pro-inflammation responses were significantly increased by buffalo milk intervention. It may be that the bioactive peptides in milk occurred in the small intestine, which can improve the permeability of the intestinal barrier.

## Conclusion

In this study, mice fed by high fat diet were perfused with cow milk and buffalo milk, and body weight gain of mice was significantly decreased by cow milk and buffalo milk perfusion. The cow milk can enhance the mRNA expression of ACAA2, ACACB, and SLC27A5 genes involved in the lipid metabolism in liver tissue. Moreover, area of lipid accumulation in liver was significantly decreased by cow milk intervention. These findings provide a better understanding of the role of cow milk in fatty liver, and thus dietary supplementation of cow milk may have beneficial effects on lipid metabolism in the context of obesity.

## Data Availability Statement

The original contributions presented in the study are included in the article/Supplementary Material, further inquiries can be directed to the corresponding author.

## Ethics Statement

The animal study was reviewed and approved by the Institutional Animal Care and Use Committee of Yangzhou University.

## Author Contributions

MJ and GZ designed the whole experiment and verified the validity of experiment and checked the results. KZ, QY, and XM performed the experiment, including chemical analysis, and statistical analysis. MJ, TY, and ZM worked on the manuscript. ZC, XG, YH, and ZM participated in the experiment design and gave valuable advice. All authors have read and approved the final version of this manuscript.

## Conflict of Interest

The authors declare that the research was conducted in the absence of any commercial or financial relationships that could be construed as a potential conflict of interest.

## Publisher’s Note

All claims expressed in this article are solely those of the authors and do not necessarily represent those of their affiliated organizations, or those of the publisher, the editors and the reviewers. Any product that may be evaluated in this article, or claim that may be made by its manufacturer, is not guaranteed or endorsed by the publisher.
