# Supplementary material for: Effects of Buffalo Milk and Cow Milk on Lipid Metabolism in Obese Mice Induced by High Fat
Source: Front Nutr. 2022 Apr 26;9:841800. doi: 10.3389/fnut.2022.841800 (PMC9089190; doi:10.3389/fnut.2022.841800)
Supplement: Supplementary file 1 [file Data_Sheet_1.docx]

**Figure1.** TIC overlap diagram of QC samples in positive and negative ionization mode, n=9.

Notes: 1. TIC is the total ion chromatogram, which takes the time point as the abscissa and the sum of the intensities of all ions in the mass spectrum at each time point as the ordinate.

2. The QC sample is the same sample, and the overlapping diagram can judge the status of the instrument.


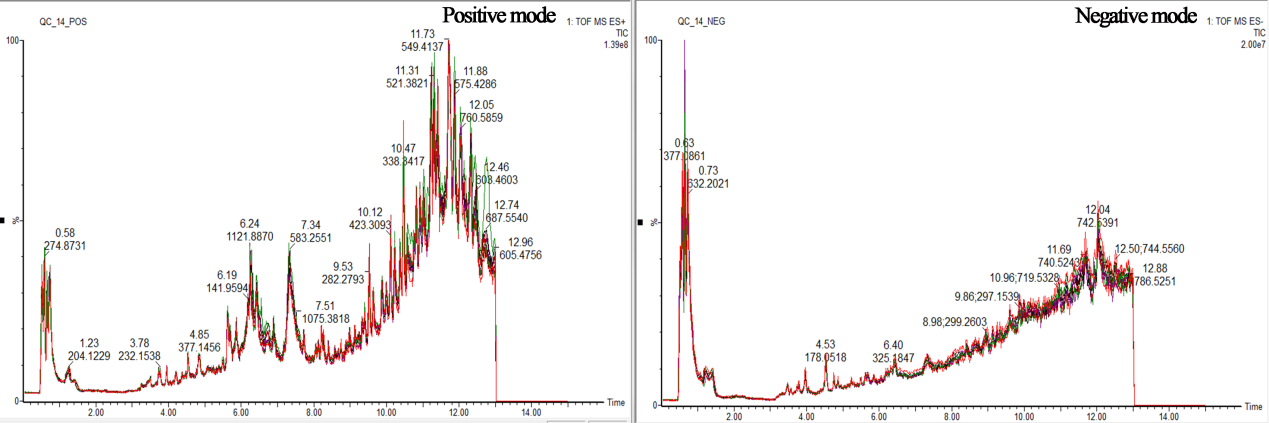


**Figure2.**  PCA scores plot of milk in buffalo and cow in positive and negative ionization mode, n=9.

Notes: The abscissa represents the first principal component PC1 and the ordinate represents the second principal component PC2, same as below.


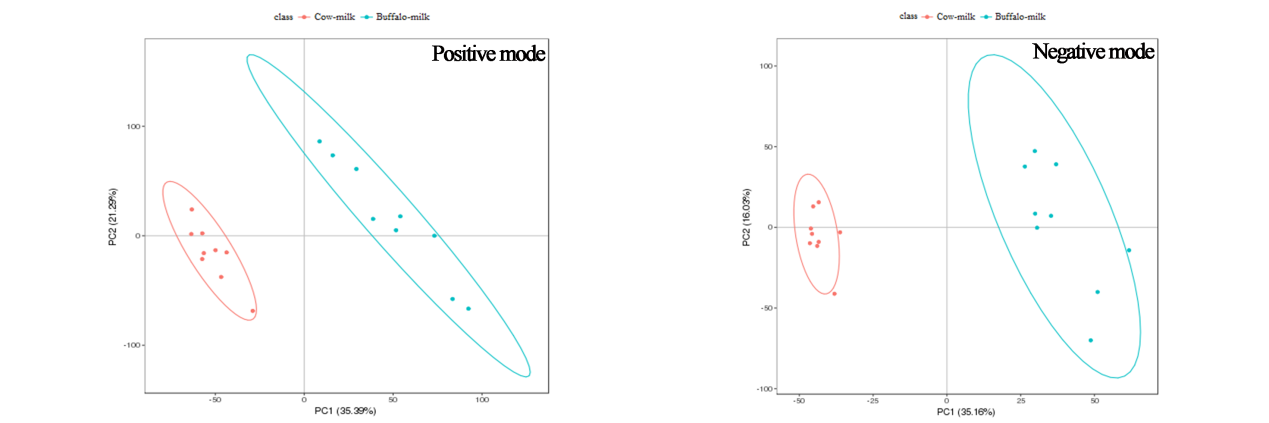


**Figure3.** PLS-DA scores plot of milk in buffalo and cow in positive and negative ionization mode, Permutation test=200.


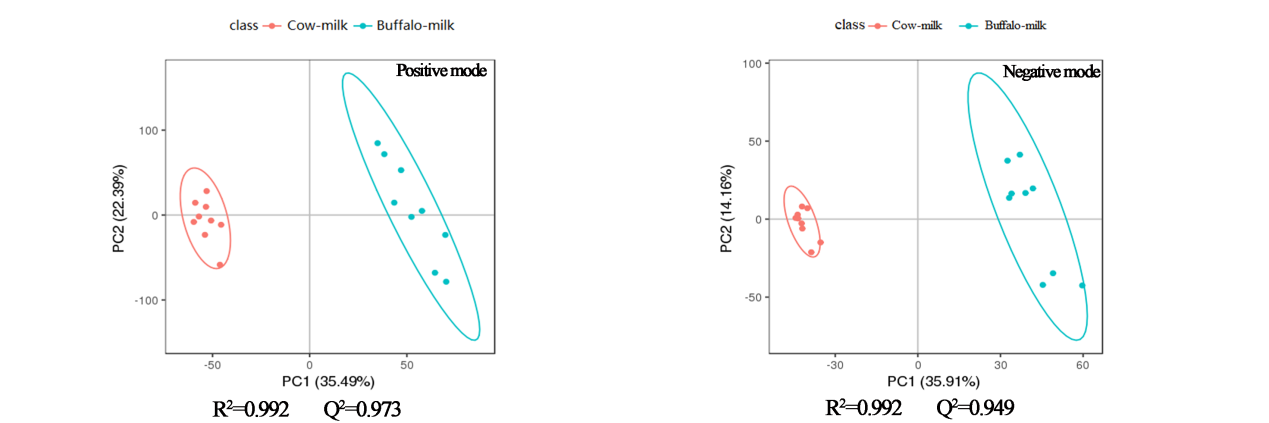


Quality control -- based robust LOESS signal correction is an effective data correction method in metabolic data analysis. The picture shows that the sample peak diagram and intensity fluctuation basically overlap in the positive and negative ion mode (Fig. 1). The results indicate that the instrument is stable for sample detection. Principal coordinate analysis (PCA) was performed to reveal differential metabolites between the two groups, and the weighted UniFrac metrics were also determined (Fig. 2). PCA in positive ion mode showed that principal coordinates 1 and 2 accounted for 35.39 and 21.29% of the total variance, and PCA in negative ion mode showed that principal coordinates 1 and 2 accounted for 32.78 and 15.08% of the total variance respectively. This indicated that the metabolite in milk from cows and buffalo were markedly distinct. By PLS-DA analysis, the model quality parameters in positive and negative ion modes were R^2^=0.992, Q^2^=0.973, R^2^=0.992 and Q^2^=0.949 (Fig. 3). In this experiment, R^2^ and Q^2^＞0.5 indicate that the model is successfully established.
